# Stability of SARS-CoV-2 on critical personal protective equipment

**DOI:** 10.1038/s41598-020-80098-3

**Published:** 2021-01-13

**Authors:** Samantha B. Kasloff, Anders Leung, James E. Strong, Duane Funk, Todd Cutts

**Affiliations:** 1 National Microbiology Laboratory, Public Health Agency of Canada, 1015 Arlington Street, Winnipeg, MB R3E 3R2 Canada; 2 Department of Pediatrics and Child Health, College of Medicine, Faculty of Health Sciences, University of Manitoba, Winnipeg, MB Canada; 3 Department of Infectious Diseases and Medical Microbiology, College of Medicine, Faculty of Health Sciences, University of Manitoba, Winnipeg, MB Canada; 4 Department of Anaesthesia and Medicine, College of Medicine, Faculty of Health Sciences, University of Manitoba, Winnipeg, MB Canada

**Keywords:** Virology, SARS-CoV-2, Policy and public health in microbiology

## Abstract

The spread of COVID-19 in healthcare settings is concerning, with healthcare workers representing a disproportionately high percentage of confirmed cases. Although SARS-CoV-2 virus has been found to persist on surfaces for a number of days, the extent and duration of fomites as a mode of transmission, particularly in healthcare settings, has not been fully characterized. To shed light on this critical matter, the present study provides the first comprehensive assessment of SARS-CoV-2 stability on experimentally contaminated personal protective equipment (PPE) widely used by healthcare workers and the general public. Persistence of viable virus was monitored over 21 days on eight different materials, including nitrile medical examination gloves, reinforced chemical resistant gloves, N-95 and N-100 particulate respirator masks, Tyvek, plastic, cotton, and stainless steel. Unlike previous reports, viable SARS-CoV-2 in the presence of a soil load persisted for up to 21 days on experimentally inoculated PPE, including materials from filtering facepiece respirators (N-95 and N-100 masks) and a plastic visor. Conversely, when applied to 100% cotton fabric, the virus underwent rapid degradation and became undetectable by TCID_50_ assay within 24 h. These findings underline the importance of appropriate handling of contaminated PPE during and following use in high-risk settings and provide interesting insight into the potential utility of cotton in limiting COVID-19 transmission.

## Introduction

Since its emergence in China at the end of 2019, the unprecedented spread of SARS-CoV-2 has led to the declaration of a pandemic by the World Health Organization. The role of healthcare settings in the spread of this virus is of particular concern, as healthcare workers have represented a disproportionately high number of confirmed cases in the first months of the COVID-19 pandemic^1–3^. Similar trends were noted in the 2003 SARS outbreak, where healthcare workers accounted for approximately 20% of cases^4^.

Among the many questions yet to be adequately addressed about the modes of transmission of COVID-19 are those related to fomite spread and environmental contamination. In healthcare settings in particular, numerous surfaces ranging from door handles to disposable gowns and gloves may become contaminated by droplets of infectious secretions and shed by infected persons. Further, global shortages in personal protective equipment (PPE) may result in items intended for single use being reused or worn for longer periods than recommended^5^. This in turn may increase the opportunity for viral contamination and subsequent spread. An understanding of the environmental stability and persistence of SARS-CoV-2 on contaminated PPE may have profound impacts on the handling of both reusable and single-use items during use and after wear.

The present study aimed to determine the stability of SARS-CoV-2 on experimentally inoculated surfaces of PPE widely encountered by healthcare workers and the members of the general population. Our findings underline the importance of appropriate handling of contaminated PPE after its use and provide interesting insight into the potential utility of cotton masks in limiting COVID-19 transmission.

## Materials and methods

### Cell culture and virus

Passage 3 stocks of SARS-CoV-2 (*hCoV-19/Canada/ON-VIDO-01/2020, GISAID accession# EPI_ISL_425177*) were prepared in Vero E6 cells as previously described^6^. Endpoint titration of stock virus and all experimental eluates were calculated via the 50% Tissue Culture Infectious dose assay (TCID_50_) on Vero E6 cells in a 96 well plate format using the method of Reed and Muench^7^. As SARS-CoV-2 is classified as a Risk Group 3 pathogen, all experimental procedures took place in a high containment laboratory at the National Microbiology Laboratory in Winnipeg, Canada.

### Test surfaces

Eight materials, representing a range of environmental surfaces commonly encountered in healthcare facilities with a particular focus on personal protective equipment were included in this study. These included nitrile medical examination gloves, reinforced chemical resistant gloves, N-95 and N-100 particulate respirator masks, Tyvek coveralls, plastic from face shields, heavy cotton, and stainless steel (Table 1).

**Table 1:** Comprehensive descriptions of materials used for assessment of SARS-CoV-2 environmental persistence.

Test Material	Description	Manufacturer/Source	Porosity
Stainless Steel	Stainless Steel (10 mm, AISI 430)	Klassen Manufacturing, Winnipeg Canada	Non-porous
Plastic face shield	Plastic face shield from PAPR Hood Assembly (BE-10L)	3 M, Saint Paul, Minnesota	Non-porous
Nitrile gloves	Nitrile Gloves (KC55081)	Kimberly-Clark Professional™, Irving, Texas	Non-porous
Chemical gloves	Chemical resistant, reinforced nitrile gloves (AlphaTec 39-124)	Ansell, Melbourne, AUS	Non-porous
Tyvek	Tyvek 400 Coveralls (TY125SWH)	DuPont, Wilmington, Delaware	Porous
Mask 1	N95 Particulate Filter Respirator and Surgical Mask (KC46727)	Kimberly-Clark Professional, Irving, Texas	Porous
Mask 2	N-100 Particulate Respirator (3M8233)	3 M, Saint Paul, Minnesota	Porous
Cotton	Heavy Cotton T Shirt	Fruit of the Loom, Bowling Green, Kentucky	Porous

Pre-cut, 1 cm^2^ stainless steel coupons were obtained directly from the manufacturer and sterilized as previously described^8^. Coupons of all other experimental materials were cut onsite to 1.4 cm^2^ and sterilized by 2 Mrads of gamma radiation prior to use.

### Experimental inoculation and sampling

Experimental inoculum consisted of stock virus (titre = 7.88 LogTCID_50_/mL) prepared in a tripartite soil load as per ASTM standard^9^ containing mucin (Sigma #M3895), bovine serum albumin (Sigma #A1933) and tryptone (Sigma #T7293) to represent the organic components of typical virus-containing fluids shed by infected individuals^10^. Ten microliters of the resulting virus suspension were added via positive displacement pipette to the center of each coupon and left to air dry in a biosafety cabinet for 1 h. Once dry, coupons were placed in a vented plastic container and stored in a closed cabinet at ambient temperature for the duration of the study. Daily recordings of temperature and humidity at 12-h intervals were monitored with a Professional Thermo-Hygrometer with data logger (TFA Dostmann Product#30.3039.IT).

Three biological replicates (N = 3) of each test material were sampled at the following time points post inoculation: T = 1 h (point of drying), 4 h, and at 1, 2, 3, 4, 7, 14 and 21 days. Two additional short-course studies on cotton were also included, using the same inoculation and sampling regime up to 3 dpi. Virus was recovered by elution of each coupon in 1 mL of culture medium (DMEM + 2% FBS + 1% Pen-Strep) with rigorous and repeated pipetting. Eluates were immediately tenfold serially diluted for endpoint titration in Vero E6 cells, with remaining volumes of eluates stored at − 80 °C for potential re-processing if required. Samples resulting in low-level cytopathic effect (i.e., in only a single well at neat dilutions) underwent additional sub-passage to confirm the presence of viable virus. Additionally, on the first instance where all biological replicates of a given sample material resulted as negative, supernatants from the neat dilutions of inoculated Vero E6 cells underwent additional sub-passage to ensure capture of potentially low-level virus not visually detected on the first passage. At the completion of the study, the full remaining volume from each day 21 eluate was added to an individual well of a 6-well plate of Vero E6 cells and monitored for cytopathic effect to maximize viable virus detection.

### Real-time RT-PCR

Real-time RT-PCR (qRT-PCR) was performed on samples collected at T = 1 h (point of drying), 4 h, and 1, 7, and 14 dpi to confirm successful elution from all surfaces and to assess differences in residual RNA compared to infectious particles. Briefly, supernatants were pooled from the three biological replicates of individual materials harvested at a given time point, and viral RNA was extracted with the Viral RNA Mini kit (Qiagen, Germany). The qRT-PCR was run on a LightCycler 96 (Roche, Germany) using the EXPRESS One-Step Superscript RT-qPCR Universal Kit (Invitrogen, USA) with primers and probes targeting the nucleocapsid (NP) gene^11^. Thermal cycling conditions were 50 °C for 15 min for reverse transcription, followed by 95 °C for 20 s and then 40 cycles of 95 °C for 3 s, 60 °C for 30 s. Values are reported as log genome equivalents per mL based on CT values obtained with a standard curve of known concentration plasmid DNA encoding for the target nucleocapsid region.

### Statistical analyses

Graphical representations of TCID_50_ results, including averages and standard deviations, were performed using GraphPad Prism (version 7) software. Biological replicates with no recoverable virus were assigned a value of zero for the purposes of these calculations.

## Results

The environmental persistence of SARS-CoV-2 dried onto surfaces of commonly used PPE and surfaces found in healthcare settings over a period of 21 days revealed dramatic differences in virus stability. By 4 h post-deposition on cotton, infectious SARS-CoV-2 virus was drastically diminished, and no longer quantifiable by 24 h. Conversely, while reduced almost to the limit of detection, SARS-CoV-2 remained viable for up to 21 days when dried on plastic and particulate respirator procedural masks. Real-time monitoring of ambient conditions revealed a steady temperature near 20 °C with 35–40% relative humidity.

### Persistence of SARS-CoV-2 on non-porous surfaces

**Figure 1:**
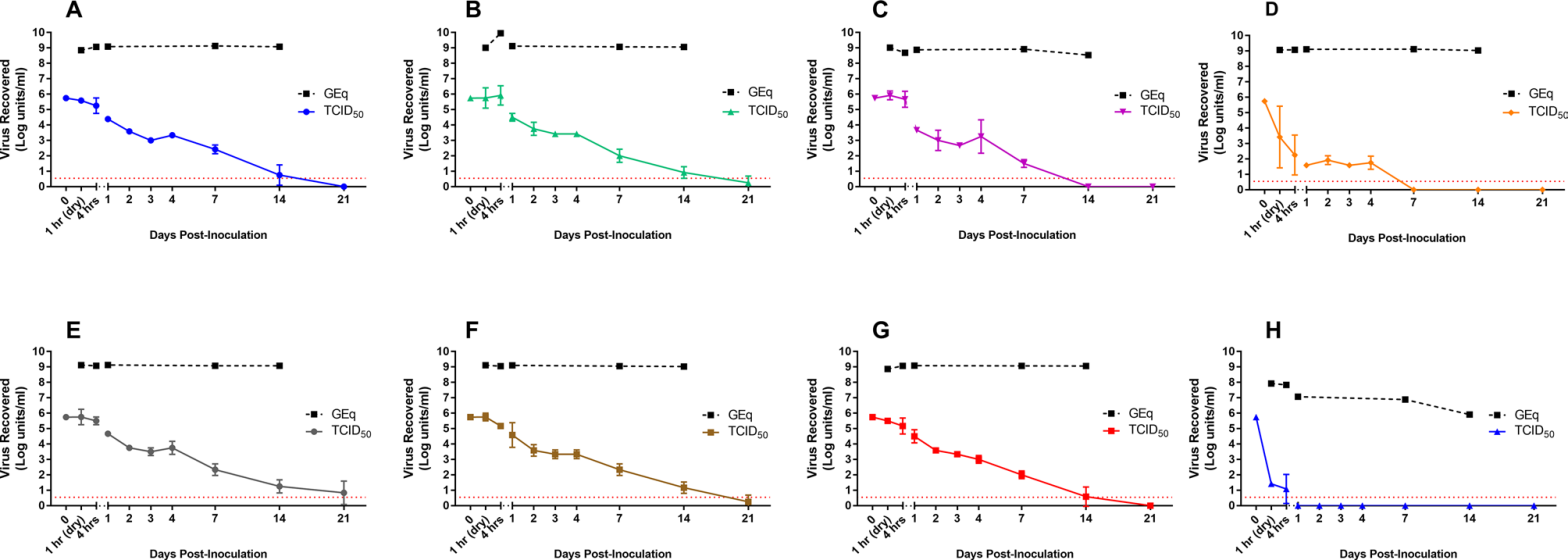
Persistence of SARS-CoV-2 on experimentally inoculated surfaces. Materials from eight experimental surfaces, including (**A**) stainless steel, (**B**) plastic, (**C**) nitrile gloves, (**D**) chemical gloves, (**E**) N95 mask, (**F**) N100 mask, (**G**) Tyvek, and (**H**) cotton were inoculated with 10 µl of SARS-CoV-2 (titre = 7.88 LogTCID_50_/mL) in a tripartite soil load and sampled at predetermined intervals over a 21 day time-course. TCID_50_ values (solid lines) represent mean ± SD of viable virus recovered from three biological replicates following end-point titration in Vero E6 cells. Genome equivalents (dashed black lines) are based on nucleoprotein-specific qRT-PCR results obtained from pooled eluates of three biological replicates at indicated sampling points. Red dashed lines indicate limits of quantification for the TCID_50_ assay.

When a dried onto non-porous surfaces in an organic soil load SARS-CoV-2 showed sustained persistence for prolonged periods of time. Viable SARS-CoV-2 was recovered after 21 days on plastic, 14 days on stainless steel, 7 days on nitrile gloves and 4 days on chemical resistant gloves, though at significantly reduced levels compared to the initial inoculum (Fig. 1).

Persistence data on stainless steel showed a decrease in viral titre from 5.74 to 4.39 log TICD_50_/ml after a 24 h, representing a > 95% decrease in viable virus. Sampling over a 21-day time course revealed that SARS-CoV-2 remained viable on stainless steel for up to 14 days at ambient conditions, though final titers of 0.7 Log TCID_50_/ml were exponentially lower than starting inoculum (Fig. 1A). On plastic, a similar reduction in viable virus was observed by 24 h of drying compared to initial inoculum (from 5.74 to 4.5 log TCID_50_/ml). However, virus dried on this surface remained viable through the final sampling point at 21 days post-inoculation (Fig. 1B).

Two glove types, representing those used in clinical and potential field hospital situations, were also included for analysis. With nitrile gloves, no decrease in viable virus was observed at 4 h post inoculation, though by 24 h a 2-log reduction in viral titer was recorded. At 7 days post-inoculation, low levels of viable virus (mean 1.50 log TCID_50_/ml) remained on all three biological replicates (Fig. 1C). Conversely, virus applied to the chemical protective gloves showed a 2-log reduction in viability after only a single hour of drying, and a nearly 4-log reduction to 1.58 log TCID_50_/ml by 24 h (Fig. 1D).

### Persistence of SARS-CoV-2 on porous materials

Though reduced to almost undetectable levels, viable SARS-CoV-2 could be recovered from inoculated N-95 (Mask 1) and N-100 (Mask 2) surface materials for up to 21 days. Decay patterns of virus viability were comparable on the two masks; both showing a nearly 1 log decrease from 24 to 48 h, stable titres between 2 and 4 days, and a steady decline from days 7 through 21 (Fig. 1E,F). On Tyvek, infectious SARS-CoV-2 persisted up to 14 days; again, reduced to very low levels compared to the starting inoculum. Persistence patterns on this material (Fig. 1G) most closely resembled those for stainless steel.

Of all the materials tested, cotton provided the lowest environmental stability to SARS-CoV-2. After only a single hour of drying, over 4 logs of viable virus were lost, representing a 99.995% decrease from input inoculum. After 4 h of drying, a further decrease was recorded and only two of three technical replicates produced viable virus upon elution. By the 24 h time point and at all subsequent sampling days, infectious SARS-CoV-2 on cotton was below limits of detection by TCID_50_ (Fig. 1H).

**Figure 2:**
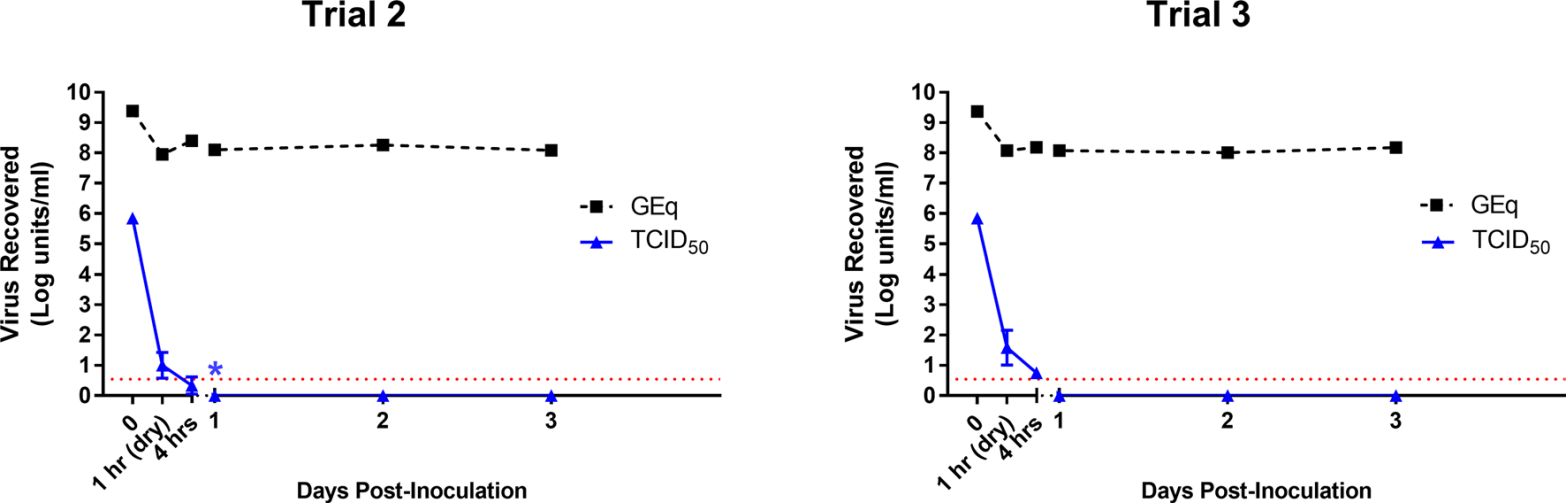
Persistence of SARS-CoV-2 on cotton. Ten microliters of SARS-CoV-2 (titre = 7.88 LogTCID_50_/mL) prepared in a tripartite soil load were inoculated onto gamma-irradiated coupons of cotton fabric and sampled at predetermined intervals over a 3-day time-course. TCID_50_ values (solid lines) represent mean ± SD of viable virus recovered from three biological replicates following end-point titration in Vero E6 cells. Genome equivalents (dashed black lines) are based on nucleoprotein-specific qRT-PCR results obtained from pooled eluates of three biological replicates at indicated sampling points. Red dashed lines indicate limits of quantification for the TCID_50_ assay. Asterisk indicates detection of viable virus in safety test of a single biological replicate.

To provide additional confidence to stability data on cotton, two additional short-term persistence trials were carried out, with sampling at identical intervals as the initial long-term experiment but up to only 3 dpi (Fig. 2). Results obtained were in agreement with those observed on the initial experiment; a single hour of drying resulted in over four logs of inactivation and additional loss of viability was recorded by 4 h post-inoculation. By the 24 h sampling point, all biological replicates from both independent experiments were negative by TCID_50_ assay. A single biological replicate from one of the trials, however, resulted in CPE on the safety test following addition of the full volume of residual inoculum (~ 500 µl) from the eluted coupon. At the time of inoculation, potential wicking of the inoculum from the cotton coupon onto the culture plate below was noted for that particular biological replicate, however this cannot be confirmed as the cause for maintained viability. No further positivity was observed at any subsequent sampling point.

### Persistence of viral RNA

To ensure complete elution from all test materials and provide insight on the persistence of viral genetic material compared with that of viable virus, qRT-PCR was performed on RNA was extracted from all eight test surfaces at various time points up to 14 dpi. In contrast with viability results, SARS-CoV-2 RNA showed high-level stability across nearly all surfaces up to the 14 day time point, with 8–9 log genome equivalents detected despite significant decreases in corresponding TCID_50_ values (Fig. 1). When eluted from cotton, SARS-CoV-2 RNA was recovered at slightly lower yet relatively high titers (~ 7.9 logs) at the 1 and 4 h sampling times, followed by a gradual reduction between days 1 and 14 post inoculation (Fig. 1H).

## Discussion

The COVID-19 pandemic has led to unprecedented burdens on healthcare facilities worldwide. While respiratory droplets are considered an important mode of transmission, the role of fomites in the spread of SARS-CoV-2, as suggested for SARS-CoV-1^12^, is also suspected^13^. Our work represents the first comprehensive characterization of SARS-CoV-2 persistence on PPE materials and inanimate surfaces typical of healthcare facilities.

Viable SARS-CoV-2 was recovered after 21 days on plastic, 14 days on stainless steel, 7 days on nitrile gloves and 4 days on chemical resistant gloves. Though reduced from baseline, significant quantities of viable SARS-CoV-2 could be recovered from inoculated N-95 and N-100 masks at 14 days. When dried onto Tyvek, infectious SARS-CoV-2 persisted up to 14 days. Of all the materials tested, cotton provided the lowest environmental stability to SARS-CoV-2. After only a single hour of drying, over 4 logs of viable virus were lost, representing a 99.995% decrease from input inoculum. These results have direct relevance to Infection Prevention and Control practices, laundering and waste handling protocols in healthcare settings.

The global shortage of N-95 masks has led healthcare centers worldwide to extend the usage of these masks despite them being designed for single use^5^. Our results demonstrate that this decision, in the absence of a decontamination strategy, may result in persistence of SARS-CoV-2 on the mask. Persistence of other viruses, such as influenza, Ebolavirus, and other coronaviruses, has been reported on experimentally-inoculated respirators^6,14,15^. While a number of decontamination methodologies show promise for the re-use of N-95s^16,17^, our results suggest that careful attention must be paid to their collection criteria and provision of appropriate risk-based PPE to staff involved in sorting and packaging of these masks prior to decontamination.

Our finding that SARS-CoV-2 can remain viable for up to 2 weeks at room temperature on Tyvek is novel and significant. Tyvek garments have been widely adopted in high-risk healthcare, field, and laboratory settings due to unique tensile properties combined with imperviousness to infectious agents. Viral persistence on Tyvek, combined with our demonstration of viral persistence on plastic has implications for the re-use of Powered Air Purifying Respirator hoods (PAPRs) in health care settings. Disinfection of these devices has proven to be challenging^18^. These results highlight the need for risk-based decision-making and implementation of appropriate decontamination protocols where re-use of these critical PPE components is practiced.

The extremely limited viability of SARS-CoV-2 on cotton aligns with survival results of other enveloped viruses on cotton, including those of human and avian origin^6,19,20^. Previous investigations by our group on the persistence of Ebola virus on PPE showed comparable trends, with viable virus decreasing by 99% within a single hour of deposition on cotton and becoming entirely undetectable by 24 h while RNA remained detectable throughout the course of study^21^. Similar to our results with SARS-CoV-2, the viability of SARS-CoV-1 when applied at 10^5^ TCID_50_/ml was limited to 1 h on a cotton gown compared to 24 h on a disposable gown^22^. Although there was a slight decrease in the amount of RNA recovered from the cotton fabric when compared to the other materials, this was minor when compared to the substantial loss of virus viability (complete absence by 24 h in 8/9 replicates). While it is possible that retention of the RNA and virus in the cotton may have contributed to a small portion of the lowered recovery, clearly the greater than 6 logs reduction of viable virus over the PCR reflects the fact that cotton has a significant effect on virus viability. The porous nature of the cotton fabric, increasing the surface area onto which the inoculum droplet was adsorbed and subsequently subjected to drying, is likely to augmented viral degradation thereby decreasing virus viability as well as quantification of RNA. While this manuscript was in its revision stage, a study released by Riddell and colleagues corroborated our observations on the reduced viability of SARS-CoV-2 on cotton compared to other materials^23^. Using a similarly high titer inoculum, a significant loss of infectious virus was noted after an hour of drying, though persistence was detectable up to 7 days. This difference may be attributed to the specific material attributes of the cotton cloths used in the two studies, and/or differences in protein content in the concentrated virus preparation without use of a sucrose cushion.

The use of non-medical masks to limit the spread of transmissible respiratory diseases, including COVID-19, has been contentious. While mounting evidence suggests that simple cloth masks could be effective in limiting SARS-CoV-2^24^, others have questioned the efficacy of manufactured or home-made cloth masks to effectively prevent release of viral particles from infected individuals^25,26^. Our observations in the present study are not intended for inference on the ability of cotton masks to prevent aerosol transmission of SARS-CoV-2, nor do they address filtration efficacy of cotton against the virus. However, the fact that virus viability is rapidly reduced on cotton exposure has implications for droplet transmission for both the wearer and nearby contacts, and complements the growing support of widespread cotton mask use. Further, these results suggest that the use of cotton-based fabrics in healthcare settings may present a lower risk during handling for subsequent decontamination and reuse. The use of cotton mask covers to extend the usage of N95 masks), a growing trend in major medical centers^27^, presents an excellent example of such a risk mitigation strategy.

The survival of SARS-Cov-2 on environmental surfaces has been described in two heavily cited studies^28,29^. Our results are in agreement with those reported, however viral persistence was significantly prolonged under our experimental conditions. These discrepancies are likely due to a combination of different ambient conditions, inoculum titres, and readout assay sensitivity. Indeed, in the recently published work of Riddell and colleagues^23^, extended persistence of SARS-CoV-2 for up to 28 days was observed under experimental conditions most closely resembling those utilized herein.

The work of Chin and colleagues^28^, using a 5 µl droplet of high titre stock virus (~ 7.8 log TCID_50_ units/mL), most closely resembled our inoculation conditions. The reported 65% RH in their study compared to ~ 40% RH under our experimental conditions is likely a significant contributor to the reduction in viability over time. The detrimental effects of high relative humidity on survival of other coronaviruses, including MERS and SARS-CoV-1, has been demonstrated^30–32^. Nevertheless, the availability of data specific to diverse environmental conditions is of importance to capture the realities at different geographical locations.

In the report by Van Doremalen et al.^29^, viable SARS-CoV-2 persisted up to only 3 days on stainless steel and plastic. While the reported ambient conditions more closely represented ours, the titre of infectious SARS-CoV-2 applied to test surfaces was approximately 2 logs lower than that applied in our study. The protective effect of high titre inoculum on environmental persistence has been previously shown for SARS-CoV-1^22^ and therefor the extended survival observed under our experimental design is not surprising.

An additional factor differentiating the present work from other studies is the matrix in which inoculum was prepared. Contamination in a healthcare setting can occur through droplets from an array of infectious biological fluids, therefore a standardized tripartite soil load^10^ was included in our experimental inoculum. The added stability provided by matrices to viruses under drying conditions has been demonstrated for many viruses, including SARS-CoV-1^10,33,34^. The recent findings of Riddell and colleagues, demonstrating persistence of viable SARS-CoV-2 up to 28 days on several surfaces^23^ strongly supports the role of an organic matrix combined with high titer inoculum in the extended viral persistence observed in our experimental setting.

The decision to use a high titre inoculum in our study was to represent a worst-case scenario of SARS-CoV-2 persistence on a contaminated surface. Shedding of viral RNA from COVID-19 patients varies widely based on sample type, time post-symptom onset, severity of disease, and on an individual basis^35,36^. Clinical data from mild and severe cases has revealed a range of data, with median initial viral loads of 5.11 and 6.17 log_10_ copies of viral RNA per ml in mild and severe cases, respectively^37^. In a critically ill patient, loads of up to 1.34 × 10^11^ copies per mL were detected^36^. Data on infectious virus shed in excreta from acutely ill COVID-19 patients is lacking. However, in a recently published SARS-CoV-2 pathogenesis study, peak viral loads of > 6 log_10_ TCID_50_/mL, corresponding to 11 log_10_ TCID_50_ RNA copies per mL were observed in the golden hamster model^38^. Taking these data into consideration, our experimental model would more closely demonstrate environmental contamination from a severe COVID-19 case.

A potential limitation of this study lies in testing being carried out in a high containment laboratory, where 12 air exchanges per hour may represent a high air flow not necessarily representative of most health facilities, homes and other environments. In addition, the initial hour of drying time under a biosafety cabinet would have accelerated the drying effect and may have shortened the amount of time to inactivate the virus. However, the revealed persistence of up to 21 days was significant and may be the best-case scenario associated with high airflow conditions that can occur in some health care environments.

Additionally, the implications of these results and those of others^23,28,29^ on the duration of potential fomite transmission of SARS-CoV-2 should be interpreted with caution due to the sampling methodology utilized. Viral recovery was achieved by material saturation in media, enabling recovery of extremely low levels of remaining virus. Had we employed a swabbing method to more closely mimic a casual contact scenario, a decrease in sensitivity would be expected and thus duration of viable virus detection may have been reduced. As a result, the data generated represent a worst-case scenario for potential exposure through these contaminated surfaces, though the risk of fomite transmission through casual contact can only be inferred by the presence of infectious virus at a given time point.

Our findings suggest that SARS-CoV-2 can remain infectious on contaminated PPE for extended periods under ambient environmental conditions. Conversely, they demonstrate that stability is highly reduced on cotton even within hours of contamination, and encourage the use of 100% cotton in infection prevention and control practices where fluid-resistant barriers are not required. These results underline the importance of proper handling of personal protective equipment during and following use in high-risk settings to minimize the likelihood of fomite transmission, and may assist centers in developing guidelines and protocols relating to decontamination and reuse of PPE in short supply.

## Supplementary Information

Supplementary Information.
