# Supplementary material for: Stability of SARS-CoV-2 on critical personal protective equipment
Source: Sci Rep. 2021 Jan 13;11:984. doi: 10.1038/s41598-020-80098-3 (PMC7806900; doi:10.1038/s41598-020-80098-3)
Supplement: Supplementary file 1 — Supplementary Information. [file 41598_2020_80098_MOESM1_ESM.pdf]

Stability of SARS-CoV-2 on Critical Personal Protective Equipment

Samantha B Kasloff[1*]; Anders Leung[1], James E Strong[1,2,3]; Duane Funk[4]; and Todd Cutts[1*#]

[1]National Microbiology Laboratory, Public Health Agency of Canada, 1015 Arlington Street, Winnipeg, Manitoba, R3E 3R2, Canada.

[2]Department of Pediatrics & Child Health, College of Medicine, Faculty of Health Sciences, University of Manitoba, Winnipeg, Manitoba, Canada.

[3]Department of Infectious Diseases and Medical Microbiology, College of Medicine, Faculty of Health Sciences, University of Manitoba, Winnipeg, Manitoba

[4]Department of Anaesthesia and Medicine, College of Medicine, Faculty of Health Sciences, University of Manitoba, Winnipeg, Manitoba, Canada.

[*]These authors contributed equally to this work.

[#]Corresponding Author: Todd.Cutts@canada.ca

Supplementary Table. Recovery of viable SARS-CoV-2 on experimentally inoculated environmental surfaces (Log10 $TCID_{50}$/mL)

Sampling Time	Stainless steel		Tyvek		Plastic		Nitrile gloves		Chemical gloves		Mask 1		Mask 2		Cotton	
	Mean[a]	+/- SD	Mean	+/- SD	Mean	+/- SD	Mean	+/- SD	Mean	+/- SD	Mean	+/- SD	Mean	+/- SD	Mean	+/- SD
1 hr	5.58	0.14	5.50	0.00	5.75	0.66	5.92	0.29	3.42	2.01	5.75	0.50	5.75	0.25	1.42	0.14
4 hrs	5.25	0.50	5.17	0.52	5.92	0.63	5.67	0.52	2.25	1.30	5.50	0.25	5.17	0.14	1.08	0.95
1 day	4.39	0.13	4.50	0.43	4.50	0.25	3.67	0.14	1.58	0.14	4.67	0.14	4.58	0.80	-	
2 days	3.58	0.14	3.58	0.14	3.75	0.43	3.00	0.66	1.92	0.29	3.75	0.00	3.58	0.38	-	
3 days	3.00	0.00	3.33	0.14	3.42	0.14	2.67	0.14	1.58	0.14	3.50	0.25	3.33	0.29	-	
4 days	3.33	0.14	3.00	0.25	3.42	0.14	3.25	1.09	1.75	0.43	3.75	0.43	3.33	0.29	-	
7 days	2.42	0.29	2.00	0.25	2.00	0.43	1.50	0.25	-		2.33	0.38	2.33	0.38	-	
14 days	0.75	0.66	0.67	0.63	1.01	0.24	-		-		1.26	0.42	1.17	0.37	-	
21 days	-		-		0.26*	0.44	-		-		0.83	0.76	0.26*	0.44	-	
Half Life[b]	3.625 hrs		2.86 hrs		13.34 hrs		9.873 hrs		0.4204 hrs		5.78 hrs		2.631 hrs		0.06885 hrs	

[a] Mean $TCID_{50}$ values calculated from three independent biological replicates. Limit of detection was Log(0.767) $TCID_{50}$/mL

[b] Half lives calculated using one phase exponential decay regression analysis in GraphPad Prism (version 7) software

(-) = no CPE detected in any biological replicate at any dilution of eluted materal.

* presence of viable virus confirmed upon subpassage
